# Efficient SARS-CoV-2 surveillance strategies to prevent deadly outbreaks in vulnerable populations

**DOI:** 10.1186/s12916-020-01886-2

**Published:** 2021-01-22

**Authors:** Damon J. A. Toth, Karim Khader

**Affiliations:** 1 Department of Internal Medicine, University of Utah School of Medicine, 295 Chipeta Way, Salt Lake City, UT 84132 USA; 2 Department of Veterans Affairs Salt Lake City Health Care System, Salt Lake City, UT USA; 3 Department of Mathematics, University of Utah, Salt Lake City, UT USA

**Keywords:** COVID-19, SARS-CoV-2, Surveillance, Long-term care facilities, Mathematical model

## Background

Nursing homes and other long-term care facility (LTCF) settings have borne a disproportionately large burden from the ongoing COVID-19 pandemic, as the combination of age and increased comorbidities place LTCF residents at high risk of severe disease and death [1]. The difficulty of keeping SARS-CoV-2 out of facilities increases with the prevalence of infections in the surrounding community. Even in “locked down” facilities where residents do not leave or receive visitors, facility staff in an infectious but asymptomatic or pre-symptomatic state can unknowingly transmit infection to residents for whom they provide care [2]. Transmission among LTCF staff has been well documented [3], and transmission between residents can be difficult to avoid, particularly in wards where contact restrictions are difficult or impossible to successfully implement, such as in dementia units [4].

Testing to identify infected LTCF residents or staff is an important tool for outbreak mitigation, as identified cases and their prior facility contacts can be isolated or furloughed to prevent further spread. However, LTCFs relying on symptom-based testing have still experienced explosive outbreaks with many transmissions occurring before any positive test result [5]. Regular surveillance testing of asymptomatic individuals could be an effective strategy to identify and interrupt “silent” outbreaks earlier. However, because frequent testing can pose a substantial financial and logistical burden, it is critical that a surveillance strategy be efficient, to maximize the risk-reduction benefit of each test.

## Main text

In the absence of empirical data to compare different surveillance schemes, mathematical modeling can provide much needed insights on the implications of different options for targeted surveillance, testing frequency, and test types. Smith et al. [6] sought to identify optimal testing strategies through the analysis of simulated LTCF outbreaks. Their stochastic model simulated LTCF resident and staff interactions based on contact data from prior work employing wearable sensors [7] and quantified transmission and disease progression from COVID-19 literature. Their simulation results in the absence of surveillance were consistent with important features of observed outbreaks, including silent introductions of SARS-CoV-2 leading to large outbreaks and realistic resident-to-staff infection ratios.

Surveillance tests were assumed to have a 24-h delay from swab to result, and test sensitivity was assumed to vary over the course of infection. Surveillance strategies varied according to who received tests and with what priority. Strategies included (i) testing targeted to individuals at LTCF admission or with symptoms, (ii) random testing among residents, staff, or all individuals, (iii) testing cascades, and (iv) group testing. Testing cascades and group testing prioritized those with severe COVID-like symptoms to reflect their clinical priority. Remaining tests subsequently “cascaded” to lower-priority individuals or as a single group test. For group testing, clinical specimens from up to 32 individual swabs were pooled together and tested as one. The different surveillance strategies were evaluated using several metrics, including efficacy in reducing outbreak size at the time of first detection and resource-efficiency. Sensitivity analyses assessed robustness to uncertainty in SARS-CoV-2 importation rate, LTCF size and structure, transmissibility, and diagnostic sensitivity of RT-PCR.

They found that testing residents and staff with any symptoms, not only those indicating severe COVID-19 disease, can dramatically improve outbreak detection, supporting expansion of testing criteria in LTCFs to include individuals with less common symptoms. In settings with ample testing resources, testing cascades were favored, with the most effective cascades prioritizing multiple indications, including both symptom-based and admission-based screening. Compared to traditional symptom-based screening, outbreaks were detected days earlier using cascades. Additionally, cascades had the greatest probability of identifying non-symptomatic cases, a known challenge for LTCF COVID-19 surveillance. In settings with scarce resources for testing, group testing was most effective, and across all epidemiological scenarios and capacities, group testing was the most resource-efficient means to improve surveillance compared to the baseline symptom-based approach. This broadly agrees with modeling results suggesting that group testing could be cost-effective for screening in large populations [8].

Strengths of this work include the use of strongly data-based assumptions for modeling dynamic, transmission-relevant interactions between individuals, which provide a realistic representation of the heterogeneity in inter-individual contacts in LTCFs. This study had some limitations. While demographic data were incorporated, other important factors for SARS-CoV-2 transmission and COVID-19 disease trajectories (e.g., age) were not included. The generalizability of these findings is unclear, as the simulation was parameterized to a contact network at a specific LTCF that may not reflect the diversity of facilities with different levels of acuity, patient populations, and living conditions. However, given the robustness of their findings about testing strategies to several alternate assumptions for SARS-CoV-2 transmissibility and test characteristics, it is plausible that the overall conclusions are applicable to a variety of settings.

## Conclusions

The results of this work broadly reflect experiences around the world: that LTCFs are susceptible to large COVID-19 outbreaks even when symptom-based testing and contact restrictions are implemented. This vulnerability is likely to remain for some time, even with LTCF staff being highly prioritized for receiving the first COVID-19 vaccines, as successful vaccine administration will pose significant logistical challenges, particularly in remote locations [9]. Increasing testing capacity and expanding surveillance beyond symptom-based screening could allow for earlier outbreak detection, facilitating timely intervention to limit transmission and save lives. The finding that surveillance testing cascades are efficient for detecting emerging outbreaks in facilities with ample resources is relevant and useful for many areas that have increased testing capacity. For regions and facilities where testing resources remain limited, pooled group testing is preferable, being both more effective and resource-efficient than cascades and other strategies.

It would be beneficial for the modeling community to further test and expand on these findings, including investigating the utility of alternatives such as rapid antigen testing [10] and the potential synergistic effects of combining surveillance testing with vaccination targeting and non-pharmaceutical interventions. This line of research not only has important implications for LTCF outbreak mitigation during this current pandemic, but also informs testing strategies during future outbreaks with novel or endemic pathogens that may cause silent outbreaks in LTCF settings.

## Data Availability

Not applicable.
